# The Eag potassium channel as a new prognostic marker in ovarian cancer

**DOI:** 10.1186/1746-1596-5-78

**Published:** 2010-12-07

**Authors:** Viren Asher, Raheela Khan, Averil Warren, Robert Shaw, Gerhard V Schalkwyk, Anish Bali, Heidi M Sowter

**Affiliations:** 1 Department of Obstetrics and Gynaecology, School of Graduate Entry Medicine and Health, Royal Derby Hospital, Uttoxeter Road, Derby DE22 3DT, UK; 2 Department of Pathology, Royal Derby Hospital, Uttoxeter Road, Derby DE22 3NE, UK; 3 Department of Gynaecological Oncology, Royal Derby Hospital, Uttoxeter Road, Derby DE22 3NE, UK; 4 Dept of Biology, Forensics and Sport, University of Derby, Kedleston Road, Derby DE22 1GB

## Abstract

**Background:**

Ovarian cancer is the second most common cancer of the female genital tract in the United Kingdom (UK), accounting for 6% of female deaths due to cancer. This cancer is associated with poor survival and there is a need for new treatments in addition to existing chemotherapy to improve survival. Potassium (K^+^) channels have been shown to be overexpressed in various cancers where they appear to play a role in cell proliferation and progression.

**Objectives:**

To determine the expression of the potassium channels Eag and HERG in ovarian cancer tissue and to assess their role in cell proliferation.

**Methods:**

The expression of Eag and HERG potassium channels was examined in an ovarian cancer tissue microarray. Their role in cell proliferation was investigated by blocking voltage-gated potassium channels in an ovarian cancer cell line (SK-OV-3).

**Results:**

We show for the first time that high expression of Eag channels in ovarian cancer patients is significantly associated with poor survival (P = 0.016) unlike HERG channel expression where there was no correlation with survival. There was also a significant association of Eag staining with high tumour grade (P = 0.014) and presence of residual disease (P = 0.011). Proliferation of SK-OV-3 cells was significantly (P < 0.001) inhibited after treatment with voltage gated K^+ ^channel blockers.

**Conclusion:**

This novel finding demonstrates a role for Eag as a prognostic marker for survival in patients with ovarian cancer.

## Introduction

Ovarian cancer is the second most common malignancy of the female genital tract in the UK. Cancer statistics from 2007 reveal that 4,317 UK women died from ovarian cancer, accounting for around 6% of all female deaths from cancer [1]. Despite advances in chemotherapy, ovarian cancer mortality rates in the UK since the early 1970 s, have remained stable at ~10-12 per 100,000 women. This is in part due to the asymptomatic nature of the disease with most women presenting at a late stage [1]. Current treatment with platinum based chemotherapy results in clinical remission in 75% of patients but the median progression free survival is only 16 to 21 months [2]. Thus, there is a clear need for the development of novel therapies to improve conventional treatments and identify new prognostic markers for survival.

Ion channels are pore-forming proteins that help establish and control voltage gradients across the plasma membranes of all living cells by allowing the flow of ions down their electrochemical gradient [3]. Voltage gated potassium (K^+^) channels have recently generated great interest due to their involvement in cell proliferation in various cancers [4]. Moreover, K^+ ^channel blockers have been shown to inhibit proliferation of the ovarian cancer cell line A2780 [5], identifying voltage-gated K^+ ^channels as potential therapeutic candidates for the treatment of cancer.

Four main K^+ ^channel subtypes (Kv1.3, K2p9.1, Eag and HERG) are found to be overexpressed in a number of tumour types [4]. K^+ ^channels have been suggested to be involved in cancer through the action on membrane potential and regulation of cell volume [6]. Hyperpolarisation of the cancer cells mediated by K^+ ^channels not only leads to increased Ca^2+ ^influx [7] a well known factor for regulation of cell proliferation but also maintains the driving force for Na^+ ^dependent nutrient transport and influencing intracellular pH [6]. K^+ ^channels have also been shown to affect cell proliferation due to their regulation of intracellular concentration of solute involved in DNA synthesis or activating a cell cycle regulating protein through the effect on cell volume, in fact rat glioma cells show optimal proliferation in a small range of cell volume [8].

Eag (Ether-a-go-go, Kv10.1) was first isolated from the fruitfly *Drosophilia melanogaster *as the leg shaking phenotype induced under ether anesthesia [9]. Eag has a restricted distribution limited to the central nervous system [10] and expressed transiently in myoblasts [11]. Chinese Hamster Ovary (CHO) cells transfected with the Eag gene show increased proliferation, growth factor independence and loss of contact inhibition compared to normal CHO cells [12]. Implantation of Eag-transfected cells in severe combined immune deficient mice resulted in tumour formation. Eag expression has also been detected by RT-PCR in cell lines from different organs including as He-La (carcinoma of cervix), SH_SY5Y (neuroblastoma) and various mammary cell lines (COLO-824, EFM-19, BT_474). Inhibition of Eag expression in EFM-19, HeLA, MCF-7, and SH-SY5Y cell lines with antisense oligonucleotides reduced their growth, demonstrating a role for Eag in cell proliferation [12]. Eag channel expression has also been demonstrated in various clinical tumours [10] and cervical cancer [13].

HERG (Human Ether-a-go-go related gene), also belonging to the Eag family, plays a fundamental role in cardiac excitability by regulating action potential repolarisation. It has been implicated in the molecular basis of familial Long QT 2 syndrome [14]. A potential role of these channels in cancer was first demonstrated in neuroblastoma cell lines [15] and they have since been described in cells lines derived from a plethora of malignant tissues and organs [16]. HERG expression has also been demonstrated in clinical cancers that include endometrial carcinoma [17], colorectal cancer [18] and acute myeloid leukaemia [19]. HERG channels are important determinants of the acquisition of an invasive phenotype in colorectal cancers [18]. These studies indicate that both Eag and HERG channels demonstrate the potential to be used as tumour markers for ovarian cancer. Further, specific blockers of these channels in conjunction with carboplatin, may be used as novel treatments in order to reduce the dosage required for treatment and associated side effects. They may also be considered as a treatment for patients with resistant or recurrent disease.

This is the first study to examine the expression of Eag and HERG channels in ovarian cancer biopsies, with subsequent relation to prognostic factors. We also sought to examine a role for Eag and HERG channels in the proliferation of ovarian cancer cells.

## Materials and methods

### Cell culture

The SK-OV-3 cell line (Passage 13) was kindly donated by Dimitra Dafou, Translational Research Laboratories, University College Hospital, London in July 2008, from an authenticated stock provided by the American Type Culture Collection (ATCC). The cell line was cultured in Dulbecco's Modified Eagle's Media (DMEM) supplemented with 10% fetal bovine serum (FBS) and antibiotics (100 u/ml penicillin and 100 μg/ml streptomycin; Invitrogen, Paisley, UK)

### Antibodies

**Figure 1:**
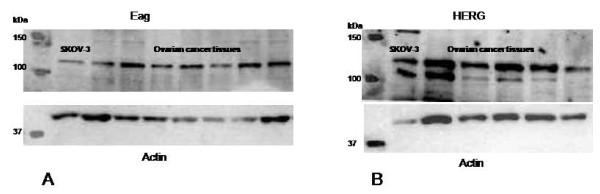
**Western blot with samples from ovarian cancer tissue and SK-OV-3 cells demonstrating specific staining for Eag and HERG antibodies (Fig 1A and B) with corresponding actin blot**.

Rabbit anti-human Eag antibody was purchased from Alomone Labs (Israel). Rabbit anti-human HERG antibody was from Abcam laboratories (UK) while secondary anti-rabbit fluorescein isothiocyanate (FITC) conjugated antibodies were obtained from Sigma (Poole UK). The antibodies were specific for the Eag and HERG protein, as demonstrated by specific staining of Eag and HERG protein in ovarian cancer tissue and SKOV-3 cells on Western blotting (Figure 1).

### Cell proliferation assay

The proliferation of SK-OV-3 cells was examined in the presence and absence of voltage gated K^+ ^channel blockers by (3-(4, 5-dimethylthiazol-2-yl)-5-(3-carboxymethoxyphenyl)-2-(4-sulfophenyl)-2H-tetrazolium, inner salt) MTS assay using the CellTiter 96 aqueous non-radioactive cell proliferation assay kit (Promega, UK). The assay was performed according to the manufacturer's instructions to assess the effect of the voltage gated K^+ ^channel blockers, 4-aminopyridine (4-AP) and tetraethylammonium (TEA) on proliferation of the SK-OV-3 cell line. 5000 cells were added to each well of a 96 well plate (Perkin Life Sciences) and incubated for 24 hours in 5% CO_2_/air at 37°C. Thereafter, the media was aspirated and replaced with 100 μl fresh media containing either 4-AP or TEA at various concentrations or media only to serve as a control. The cells were incubated for 96 hours and proliferation assessed daily by the addition of MTS (3-(4, 5-dimethylthiazol-2-yl)-5-(3-carboxymethoxyphenyl)-2-(4-sulfophenyl)-2H-tetrazolium, inner salt) reagent. Plates were then read at 490 nM using a Victor 1427 multilabel counter (Wallac). All observations were performed in triplicate and experiments repeated thrice.

### Immunofluorescence

SK-OV-3 cells were grown on cover slips in 24 well plates and incubated for 24 hours until 80% confluent. Cells were then fixed with 4% paraformaldehyde for 20 min and treated with 0.5% Igepal before blocking with 3% BSA/5% glycine in PBS and 10% goat serum in PBS. Eag and HERG (both 1: 50 and 1:100 in10% goat serum/PBS) antibodies were added to the cover slips then incubated overnight at 4°C. Wells containing 10% goat serum without the primary antibody were used as a negative control. After incubation, FITC conjugated secondary antibody at 1 in 50 dilution in 10% goat serum/PBS appropriate to Eag and HERG was added and cells incubated at room temperature for 90 min. Images of the cells were captured and analysed using Cell F software (Olympus UK).

### Immunohistochemistry

The ovarian cancer tissue microarray (TMA) (SDLREC Ref 0205/495) was prepared from 336 patients who underwent surgery for ovarian cancer from 1^st ^January 1982 to 31^st ^December 1998. Clinicopathological characteristics recorded included age at diagnosis, International Federation of Gynecologists and Obstetricians (FIGO) stage, grade, histological subtype, details of adjuvant treatment, extent of cytoreduction and Disease Specific Survival (DSS) which was then used for analysis. DSS was calculated from the date of the operation until 31 Dec 2005, when any remaining survivors were censored.

We calculated that a sample size of 336 patients allowed an 80% chance of detecting a hazard ratio of ≥ 1.40 and ≤0.71 (N Query Advisor software). The TMA were prepared by Ian Spendlove, University of Nottingham as described previously [20], and were kindly donated to us. For each tumour, 5 μM section, slides were stained with H&E to locate representative areas of viable tissue. Needle core biopsies (0.6 mm) from the corresponding areas on the paraffin embedded tumour blocks were then placed in prespecified coordinates in recipient paraffin array blocks using a manual tissue arrayer (Beecher instruments). Five copies of the array were assembled using different points within the representative tumour area. 5 μM thick sections from each TMA block were placed on coated glass slides to allow for the procedure to be performed. Thus all the biopsies in the TMA were representative of the sample and were validated.

After dewaxing in xylene, tissue sections were rehydrated in a graded alcohol series and endogenous peroxidase activity blocked by immersing the slides in 2% hydrogen peroxide in PBS for 20 min. Antigen retrieval was performed by microwaving the slides in 0.1 M sodium citrate buffer at 800 W for 15 min followed by 400 W for 10 min. After blocking with horse serum, the slides were incubated with either HERG antibody (1:200) for 1 hour at room temperature or Eag antibody (1:600) overnight at 4°C. Staining was developed using the Vectastain Elite kit and di-aminobezidine (DAB) chromagen (Dako, Hamburg, Germany) as per manufacturer's instructions. Slides were counterstained with Harris's hamatoxylin and dehydrated in alcohol before mounting in Depex for viewing.

The staining intensity was graded as: 0-no staining, 1-low, 2-intermediate and 3-high by three independent observers (VA, HS and GVS) who had no knowledge of the pathological variables or the disease outcome for the dataset.

### Statistical analysis

Analysis was performed using SPSS (version 17.0). Continuous data were analysed using median, interquartile range (IQR) and 95% confidence intervals (CI). Fisher's exact tests were used for comparative analysis of categorical data. Kaplan Meier survival estimates using log-rank testing were determined for all baseline factors specifically stage, grade, residual disease, adjuvant chemotherapy, histological type, and Eag and HERG immunostaining. In all cases, a P value of <0.05 was considered statistically significant. The cell proliferation assay data was analysed using one way analysis of variance with Dunnett's multiple comparison test (Graphpad Prism 5).

## Results

### 1. Eag and HERG potassium channels are expressed in the SK-OV-3 cell line

**Figure 2:**
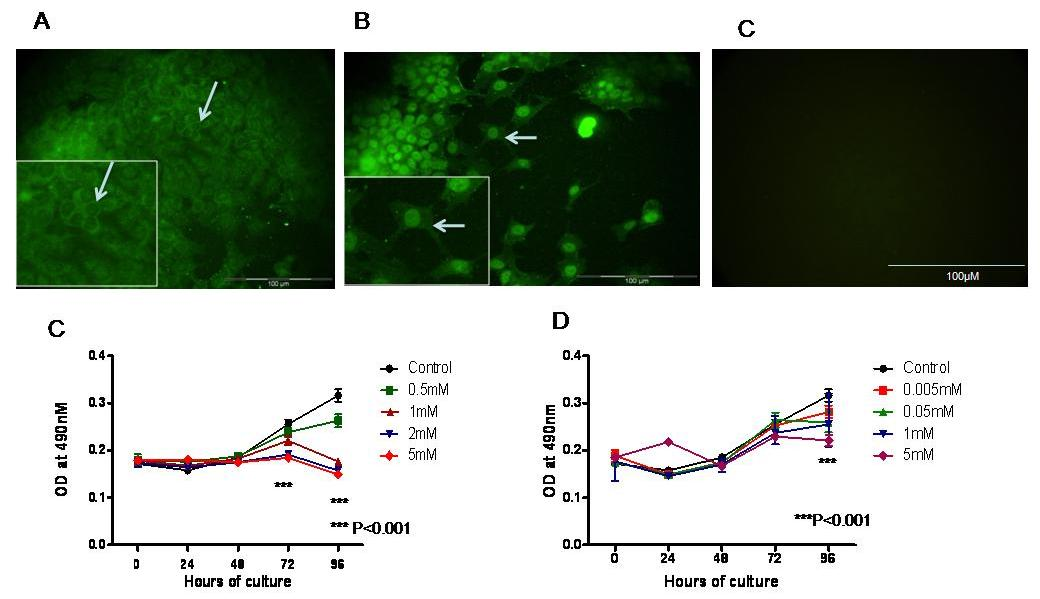
**Immunocytochemistry of SK-OV-3 cells and effect of voltage gated K+ channel blockers 4-AP and TEA on their proliferation.** Immunofluorescence staining of SK-OV-3 cells with (A) Eag and (B) HERG antibodies. Eag localises to the cell membrane and cytoplasm with little nuclear staining whereas HERG expression is mainly nuclear (higher magnification is shown in inset) (C) 4-AP significantly inhibited SK-OV-3 cell proliferation at 1,2 and 5 mM at 72 and 96 hrs while TEA (D) showed similar inhibition at 5 mM that was significant at 96 hours only.

There is demarcated staining of the plasma membrane of cells incubated with Eag antibody while nuclear staining was seen when the cells were incubated with HERG antibody (n = 3; Figure 2A and 2B, higher magnification is shown in inset). Diffuse staining of the cytoplasm and nuclei was seen with both Eag (Figure 2A) and HERG antibody (Figure 2B). No immunoreactivity was observed in negative control experiments where primary antibody was replaced with goat serum (Figure 2C).

### 2. K^+ ^channel blockers inhibit cell proliferation of SK-OV-3 cells

4-aminopyridine (4-AP) inhibited cell proliferation significantly (P < 0.001) at concentrations of 1, 2 and 5 mM compared to control cells at 96 hours with maximum inhibition noted at 5 mM (n = 3). Tetraethylammonium (TEA) inhibition of proliferating cells was also noted at all concentrations at 96 hours (n = 3; P < 0.001) (Figure 2C and 2D) compared to untreated cells.

### 3. Eag is a significant prognostic marker for ovarian cancer

**Figure 3:**
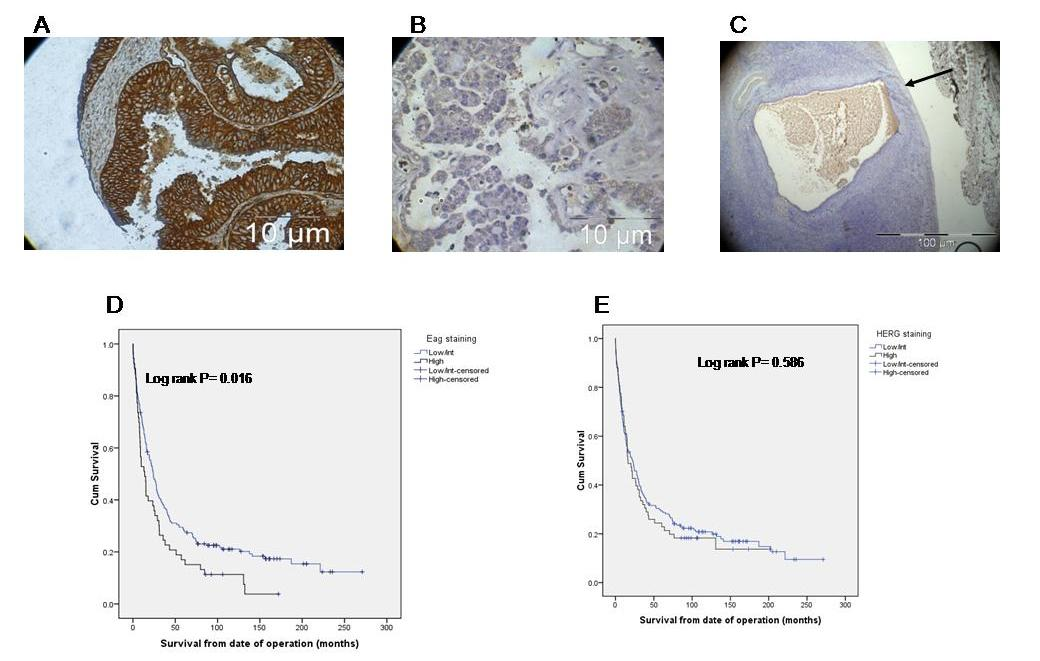
**Immunohistochemical staining of ovarian cancer tissues and normal ovary and its correlation with survival. **Representative section from a tissue microarray set showing (A) high and (B) low Eag immunohistochemical expression and low staining on the surface epithelium (arrow) of normal ovary (C). Similar results were obtained for HERG. (D) Kaplan-Meier survival curves from data represented on the above TMA show high Eag expression is associated with poor prognosis (P = 0.016), whereas increased HERG expression (E) is not.

Clinicopathological characteristics along with Eag and HERG staining of 336 patients with ovarian cancer are summarised in Table 1. The median age at diagnosis and overall survival was 62 years (IQR 24-90) and 19.5 months (IQR 0-271) respectively. Nearly half of the patients (49.4%) had FIGO stage 3 disease and two thirds (65.8%) had grade 3 tumour. The predominant histological type observed is serous (49.4%) followed by endometriod (11.7%) and mucinous (9.7%). The intensity of Eag and HERG staining was stratified into two groups of high and low/intermediate staining (Figure 3A and 3B). The survival curves for these groups are shown in Figure 3C and 3D. Biopsies with an insufficient number of cancer cells to produce a reliable result were not included in the final analysis. Patients with high Eag staining had significantly poor survival of 13.8 months (CI 7.2-20.3) compared with 24.1 months (18.9-29.2); (P = 0.016) for the low/intermediate group (Table 2). HERG staining did not show significant correlation with survival (P = 0.586). Other prognostic markers for ovarian cancer such as stage, grade, residual disease, histological type and adjuvant therapy also showed significant differences in median survival (Table 2).

**Table 1:** Patient characteristics (n = 336)

**Age at diagnosis**, years, median (IQR)	62 (24-90)
**Overall survival**, months, median (IQR)	19.5 (0-271)

**FIGO Stage**	n (%)

1	84 (25.0)

2	36 (10.7)

3	166 (49.4)

4	40 (11.9)

Missing	10 (3.0)

	

**Grade**	n (%)

G1	39 (11.6)

G2	73 (21.7)

G3	221 (65.8)

Missing	3 (0.9)

	

**Residual disease**	n (%)

No macroscopic disease (Optimal debulking)	128 (38.1)

Macroscopic disease left	194 (57.7)

Missing	14 (4.2)

	

**Histological type**	n (%)

Serous	178 (53)

Mucinous	35 (10.4)

Endometriod	42 (12.5)

Clear cell	25 (7.4)

Undifferentiated	54 (16.1)

Missing	2 (0.6)

	

**Chemotherapy**	n (%)

Yes	236 (70.2)

No	92 (27.4)

Missing	8 (2.4)

	

**Eag staining**	n (%)

Low/Intermediate	196 (58.3)

High	53 (15.8)

Missing	87 (25.9)

	

**HERG staining**	n (%)

Low/Intermediate	181 (53.9)

High	68 (20.2)

Missing	87 (25.9)

**Table 2:** Median survival based on Eag and HERG immunoreactivity and other prognostic markers.

	Median survival, Months(IQR)	P (log rank)
**Eag staining**		

Low/Intermediate	24.1 (18.9-29.2)	

High	13.8 (7.2-20.3)	0.016

		

**HERG staining**		

Low/Intermediate	22.4 (14.1-30.6)	

High	16.4 (9.7-23.0)	0.586

		

**Stage**		

1	137.6 (48.8-226.3)	

2	28.4 (4.8-51.9)	

3	13.0 (9.0-16.9)	

4	7.3 (0-15.2)	< 0.001

		

**Grade**		

G1	28.9 (17.2-40.5)	

G2	33.1 (16.4-49.7)	

G3	16.0 (13.0-18.9)	0.001

		

**Residual disease**		

No macroscopic disease (Optimal debulking)	65.2 (37.5-92.8)	

Macroscopic disease	10.7 (7.6-13.7)	< 0.001

		

**Histological type**		

Serous	17.3 (12.6-21.9)	

Mucinous	30.7 (0-64.7)	< 0.001

Endometriod	26.6 (5.3-47.8)	

Clear cell	155 (89.7-222)	

Undifferentiated	13.3(5.3-21.2)	

		

**Adjuvant** **Chemotherapy**		

Yes	20.3 (15.4-25.1)	

No	18.8 (0.6-36.9)	0.04

Following identification of a significant association of Eag staining with survival, further evaluation was carried out between the two groups of patients with Eag staining and other prognostic makers. There was no difference in the proportion of patients with stage of disease (P = 0.587), histological type (P = 0.622) or receiving adjuvant chemotherapy (P = 0.594), but there was significant correlation between high Eag staining, grade of tumour (P = 0.014) and residual disease left after the operation (P = 0.011) (Table 3). On Cox multivariate analysis (Table 4) patients with high Eag staining did not show a significant effect (P = 0.215) on overall survival compared to other prognostic markers like FIGO stage and residual disease. Staining of normal ovary (n = 6) demonstrated low level expression of both Eag and HERG on the surface epithelium compared to ovarian cancer (shown by arrow in Figure 3C).

**Table 3:** Distribution of prognostic markers for ovarian cancer stratified by Eag staining (Fisher's exact test)

Prognostic Markers	No of patients stratified by Eag staining		P value
	**Low/Intermediate**	**High**	

**Stage**			

1	52	10	0.587

2	23	7	

3	92	30	

4	23	5	

			

**Grade**			

G1	19	13	0.014

G2	44	7	

G3	133	32	

			

**Residual disease**			

No Macroscopic disease (Optimal debulking)	85	13	0.011

Macroscopic disease	102	38	

			

**Histological type**			

Serous	110	27	0.622

Mucinous	17	8	

Endometriod	24	6	

Clear cell	17	3	

Undifferentiated	28	9	

			

**Adjuvant Chemotherapy**			

Yes	144	37	0.594

No	48	15	

**Table 4:** Cox proportional hazard model of overall survival using Eag staining (Low/Int versus high) with other prognostic factors.

Variable	Hazard ratio (95% CI)	P
**FIGO Stage**		

1	1.00	

2	1.77 (1.00-3.12)	0.048

3	2.99 (1.83-4.88)	< 0.001

4	3.85 (2.02-7.34)	< 0.001

		

**Residual disease**		

No macroscopic disease (Optimal debulking)	1.00	

Macroscopic disease	1.76 (1.19-2.62)	0.005

		

**Grade**		

1	1.00	

2	1.09 (0.63-1.88)	0.756

3	1.18 (0.74-1.88)	0.474

		

**Eag staining**		

Low/Int	1.00	

High	1.25 (0.87-1.78)	0.215

## Discussion

Voltage gated K^+ ^channels have been implicated in cell proliferation and the control of cell cycle progression. They also show cell and tissue-specific expression, and impaired expression of K^+ ^channels has been detected in a number of cancer and tumour cells [21].

Both K^+ ^channel blockers, 4-AP and TEA, significantly inhibited the proliferation of Eag and HERG-positive SK-OV-3 cells, confirming data by Zhanping *et al*., on the A2780 ovarian cancer line [5]. Both TEA and 4-AP are non selective K^+ ^channel blockers capable of inhibiting Eag and HERG as well as other voltage gated K^+ ^channels. The therapeutic potential of blocking Eag and HERG channels non-specifically may be limited by cardiotoxicity toxicity resulting from impaired HERG channel activity in the heart. However, recently a monoclonal Eag antibody has been developed which specifically inhibits proliferation based on its Eag blockade with no interaction with HERG channels and has been postulated as a potential therapeutic for the treatment of cancer [22].

We show here that high Eag expression is associated with poor prognosis in patients with ovarian cancer. Hemmerlein *et al.*, have reported the presence of Eag channels in ovarian cancer tissue but no expression with normal ovarian follicular epithelium [10].

High Eag expression is also associated with high grade ovarian cancers and the presence of residual disease at surgery, which in turn is associated with poor outcome, but no correlation between tumour stage, histological type or adjuvant chemotherapy was demonstrated. In contrast to our findings, no association has been shown between Eag expression and different grades of tumour in soft tissue sarcoma patients [23] which may be due to the non epithelial origin of these tumours. Eag channels are expressed on the surface of the cell membrane are attractive targets for novel treatments in management of ovarian cancer. HERG expression did not show any difference in survival indicating that HERG channels as targets may not have a therapeutic role in ovarian cancer. Though both Eag and HERG channels belong to the same Eag family and despite blockade by the voltage gated blockers and similar positivity on staining, these two channels give rise to very different electrical currents and have different functional role in human tissues. This difference in functionality may be one of the reasons that Eag is linked to poor survival while HERG is not. This is likely to be mediated through cellular mechanisms that link to changes in membrane potential which we intend to explore further. Eag showed significant survival difference on univariate analysis, but there was no significant difference on Cox regression analysis which may be due to insufficient number of patients and having a larger cohort may also change the difference between the two markers.

## Conclusion

In summary, we have demonstrated for the first time that Eag and HERG K^+ ^channels are overexpressed in ovarian cancer and that high Eag staining is associated with significantly poorer survival, identifying Eag as a putative prognostic marker. Moreover inhibiting proliferation of ovarian cancer cells using K^+ ^channel blockers indicates that they have a role in ovarian cancer cell proliferation. Further research into specific Eag blockers or using RNA interference to silence the Eag gene is required in order to elucidate the role of Eag channels in ovarian cancer progression and their link to clinical outcome.

## Financial support

Derby Hospitals Charitable funds, Gynaecology Oncology Research Fund No 21470.

## Competing interests

The authors declare that they have no competing interests.

## Authors' contributions

VA performed the experiments and wrote the manuscript, RK,AV guided in performing experiments and helped in correction of manuscript, RS, AB helped in collection of tissue samples, GVS and HS helped in reviewing of slides and correction of manuscript. All authors read and approved the final manuscript.
